# SPP1^+^ macrophage-driven interactions shape the tumor microenvironment in lymph node metastatic acral melanoma

**DOI:** 10.1038/s41419-026-08755-5

**Published:** 2026-04-22

**Authors:** Yao Liang, Yuli Zheng, Zhimou Cai, Zhangrui Shi, Maoqia Shen, Ruixin Yu, Fang Wang, Xiaolong Cao, Rui Xu

**Affiliations:** 1 State Key Laboratory of Oncology in South China, Guangdong Provincial Clinical Research Center for Cancer, Collaborative Innovation Center for Cancer Medicine, Sun Yat-sen University Cancer Center, Guangzhou, China; 2 Department of Gastric and Melanoma Surgery, Sun Yat-sen University Cancer Center, Guangzhou, China; 3 Department of Oncology, Zhujiang Hospital, Southern Medical University, Guangzhou, China; 4 Department of Otolaryngology, The First Affiliated Hospital of Sun Yat-sen University, Guangzhou, China; 5 Department of Anesthesiology, Zhujiang Hospital, Southern Medical University, Guangzhou, China; 6 Department of Dermatology, The First Affiliated Hospital, Sun Yat-Sen University, Guangzhou, China; 7 Dermatology Hospital, Southern Medical University, Guangzhou, China; 8 Translational Medicine Research Center, Zhujiang Hospital, Southern Medical University, Guangzhou, China

**Keywords:** Cancer stem cells, Melanoma

## Abstract

Melanoma is a highly aggressive skin cancer with biologically distinct subtypes. Acral melanoma (AM), a rare but particularly aggressive form, often presents with lymph node (LN) metastasis at diagnosis. Despite its clinical severity, the mechanisms underlying its metastatic behavior remain unclear. Emerging studies suggest the tumor microenvironment as a key driver of metastatic niche formation, but its specific role in AM progression is not well characterized. To investigate the role of the tumor microenvironment in AM progression, we performed single-cell RNA sequencing (scRNA-seq) on tumor tissues and matched adjacent normal samples from treatment-naïve AM patients, comparing cases with (LN^+^) and without (LN^-^) lymph node metastasis. Key transcriptomic findings were validated by immunofluorescence staining. Functional relevance was tested by conducting in vitro and in vivo assays. An independent validation cohort was employed to confirm key observations and evaluate prognostic associations. Our results reveal preferential SPP1^+^ signaling pathways, including autocrine amplification within secreted phosphoprotein (SPP) 1^+^ macrophages and their interactions with S100A8^+^ melanoma cells via the SPP1-CD44 axis. S100A8^+^ melanoma cells emerged as the predominant malignant subpopulation in LN metastatic tumors (56.3% versus 34.7% in non-metastatic cases). Clinically, elevated SPP1 expression emerged as an independent predictor of poor overall survival. In vivo, anti-SPP1 therapy induced a macrophage phenotype switch and significantly reduced tumor burden. Together, these findings indicate that LN^+^ AM is characterized by an SPP1^+^ macrophage-driven immunosuppressive microenvironment and highlight the SPP1-CD44 axis as a promising therapeutic target for limiting AM dissemination.

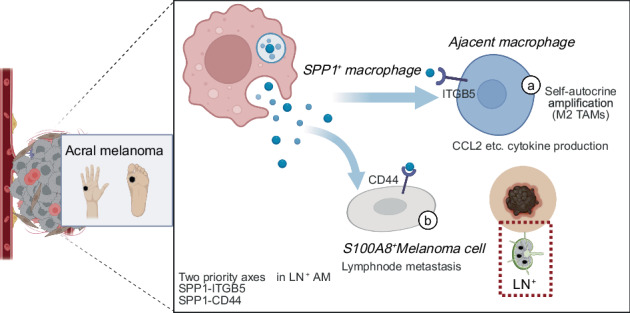

## BACKGROUND

Acral melanoma (AM) is a rare but aggressive subtype of melanoma typically developing on glabrous skin, such as the palms, soles, and nail beds [1]. In contrast to cutaneous melanoma (CM), which is driven by ultraviolet radiation and characterized by a high tumor mutational burden (TMB), AM exhibits distinct genomic and clinical profiles [2]. Driven primarily by copy number variations rather than point mutations, AM lacks sufficient neoantigens to elicit robust immune responses [3, 4]. Clinically, this manifests as an “immune desert” tumor microenvironment (TME), characterized by sparse cytotoxic T-cell infiltration and a profound enrichment of immunosuppressive subsets, including regulatory T cells (Tregs) and M2-polarized macrophages [5–7]. Consequently, AM patients exhibit notably lower response rates to immune checkpoint blockades (e.g., anti-PD-1/CTLA-4) and targeted therapies compared to CM patients, underscoring the urgent need to elucidate the unique immunobiology of AM [8, 9].

A critical driver of the aggressive clinical course in AM is early lymph node (LN) metastasis, which serves as a primary indicator of systemic dissemination and correlates with significantly reduced survival [10–14]. To date, several studies have explored the mechanisms underlying LN metastasis in AM. For instance, focal amplifications involving LZTR1 and the presence of MYC^+^ melanoma cells interacting with specific immune subsets have been linked to LN involvement [9, 15]. However, the complex cellular crosstalk orchestrating the immunosuppressive niche during this early metastatic process remains incompletely understood.

Emerging evidence highlights secreted phosphoprotein 1 (SPP1, or osteopontin) as a crucial driver of tumor progression and microenvironment remodeling. By binding to Integrins (e.g., αvβ3) and CD44 receptors, SPP1 activates PI3K/AKT and MAPK/ERK pathways, thereby promoting the epithelial-mesenchymal transition (EMT) and tumor invasion [16, 17]. Crucially, the biological function of SPP1 is highly dependent on its cellular source. While stroma-derived SPP1 primarily promotes angiogenesis, myeloid-derived SPP1 profoundly reshapes the TME into an immunosuppressive state [18, 19]. However, while elevated SPP1 expression generally correlates with poor prognosis in melanoma [20], its specific cellular origin or contribution to LN metastasis, as well as its precise interactions with metastatic tumor cells in AM, remain completely unexplored.

Here, we conducted unbiased single-cell transcriptomic profiling of skin specimens from AM patients with (LN^+^) or without (LN^-^) lymph node metastasis. Our analysis unveiled a striking enrichment of SPP1^+^ macrophages specifically in LN^+^ AM, alongside the identification of a distinct, highly aggressive melanoma cell subset (S100A8^+^). By mapping ligand-receptor interactions and inferring signaling pathways, we defined a novel immunosuppressive crosstalk between these SPP1^+^ macrophages and S100A8^+^ melanoma cells. By elucidating this SPP1-driven autocrine-paracrine axis, this study maps the TME landscape of LN metastatic AM and identifies vital therapeutic targets to improve patient outcomes.

## Methods

### Patient biopsies

This study prospectively enrolled 11 treatment-naïve AM patients (Table S1) and 25 additional patients in validation cohort (Table S2). All participants provided written informed consent in accordance with protocols approved by the Institutional Review Board of Sun Yat-sen University Cancer Center (Approval No. IRB2022-337-01).

### Animal models

For subcutaneous tumor models, 2 × 10^5^ B16F10 cells (iCell, Cat# iCell-m006) were injected subcutaneously into 8–10-week-old C57BL/6 mice (GemPharmatech Co., Ltd, China). For the spontaneous lymph node metastasis model, B16F10 cells mixed with RAW264.7 (Procell, Cat# CL-0190) macrophages were injected submucosally into the border of the tongue [21]. All mice were adults. All procedures were approved by the Institutional Animal Care and Use Committee of Sun Yat-sen University (protocol SYSU-IACUC-2024-002157) and conducted in accordance with the National Research Council’s Guide for the Care and Use of Laboratory Animals.

### Cell lines

A375 human melanoma cells (Procell, Cat# CL-0014) were maintained in Dulbecco’s Modified Eagle Medium (DMEM) supplemented with 10% fetal bovine serum (FBS) and 1% penicillin-streptomycin. B16F10 murine melanoma cells were cultured in DMEM with 10% FBS and 1% penicillin-streptomycin. THP-1 cells (a human monocytic leukemia line) (iCell, Cat# iCell-h213) were maintained in Roswell Park Memorial Institute (RPMI) 1640 medium supplemented with 10% FBS and 1% penicillin-streptomycin. RAW264.7 murine macrophage cells were cultured in DMEM supplemented with 10% FBS and 1% penicillin-streptomycin. All cultures were maintained at a density of 1–2 × 10^5^ cells/mL in a humidified incubator at 37 °C with 5% carbon dioxide (CO_2_). All cell lines were routinely tested for mycoplasma contamination, including immediately prior to experiments.

### Single-cell RNA sequencing and data processing

Single-cell sample preparation and sequencing were performed by CapitalBio Technology. Briefly, single-cell suspensions were prepared from AM specimens and assessed for viability and concentration, and then adjusted to 300–600 cells/µL. Cell suspensions were loaded on to the 10x Genomics Chromium single-cell controller to generate Gel Bead-In-EMulsions (GEMs), where cell lysis, mRNA capture, reverse transcription and barcode-labeling occurred. Libraries were constructed from the GEMs and sequenced on an Illumina NovaSeq 6000 platform with the paired-end 150 bp (PE150) reading strategy.

Raw sequencing reads were processed using the 10x Genomics Cell Ranger software (v8.0.0) and aligned to the human reference genome (GRCh38). To ensure high-quality data, we applied stringent filtering criteria to remove low-quality cells and doublets. Guided by the best practice for single-cell data analysis [22], cells were removed if identified as doublets by scDblFinder (v1.17.0) under the default settings [23]. Data were then imported into the Seurat R package (v5.1.0) [24] for further processing. Low-quality cells (less than 500 genes/cell, more than 25% mitochondrial genes, more than 40% ribosomal protein genes, or more than 10% hemoglobin genes) were eliminated when the merge function was used to combine the Single-cell RNA sequencing (scRNA-seq) data for all AM samples. This rigorous quality control process resulted in the removal of 18.0% of the initial cell population.

The scVI approach [25] was used to adjust for the possible batch effect between samples. The graph-based clustering method with the FindClusters function was used to find discrete cell groupings based on the first 10 dimensions from the scVI reduction. Major cell clusters were identified and displayed using a Uniform Manifold Approximation and Projection (UMAP) scatter plot. Cell type annotation was performed using manual refinement based on the expression of canonical cell type-specific marker genes. Tumor cells were determined using the inferCNV (v1.14.2) package [26, 27]. Cell subpopulation identities were further validated by examining the expression of known marker genes and by identifying differentially expressed genes (DEGs) that define each cluster. DEGs were identified using the FindAllMarkers function in Seurat, employing the default Wilcoxon rank-sum test.

M1 and M2 polarization scores of myeloid subpopulations were calculated using the AddModuleScore function. To assess the polarization preference of each subpopulation, we employed the ratio of observed to expected (Ro/e), defined as the observed cell numbers (actual counts) divided by the expected cell numbers calculated using the method applied in the chi-square test.

### Cell–cell interactions analysis

To elucidate potential cell-cell interactions, we utilized the CellChat (v2.1.2) package [28] and NicheNet (v2.1.0) package [29] to analyze inferred communication patterns and ligand-target regulatory relationships, respectively. We then used NicheNet to predict the receptors on SPP1^+^ macrophage cells and melanocytes that interact with *SPP1*, and the downstream target genes regulated by this interaction. We used DEGs within SPP1^+^ macrophage cells and melanocyte subpopulations as potential receptors and potential targets for NicheNet analysis, focusing on the two interaction pairs previously identified.

### Pathway enrichment analysis

As mentioned previously, the FindMarkers function within the Seurat package was used to identify DEGs within each cell subpopulation. Enrichment analyses were then performed using the Gene Ontology (GO), Kyoto Encyclopedia of Genes and Genomes (KEGG) and Reactome pathway databases. Gene Set Enrichment Analysis (GSEA) was performed using the R package fgsea (v1.24.0), with genes ranked according to their average log2 fold change (log2FC) values. For each gene set, the Normalized Enrichment Score (NES) was calculated, and the significance of enrichment was determined using a permutation test with 10,000 iterations.

### Public sequencing data processing

Bulk RNA-seq data were obtained from the CRC cohort published by Farshidfar et al. [15]. with GEO accession GSE162682. This dataset consists of gene expression values for 21 AM patients. These transcript-per-million (TPM) values were used directly for survival and prognostic analysis without further normalization. scRNA-seq data published by Hengkang Liu et al. [30]. were downloaded and analyzed similarly to data of current study, and was used to compare *SPP1* expression between LN^-^ and LN^+^ tumor samples.

### Immunofluorescence (IF) staining

Tissue microarray (TMA) sections (4–5 µm) were deparaffinized in xylene and rehydrated through graded ethanol, followed by antigen retrieval using citrate or Tris-EDTA buffer (pH 6.0) via heat treatment. The sections were blocked with 5% goat serum for 1 h. The sections were then incubated overnight at 4 °C with primary antibodies, including rabbit anti-Osteopontin (SPP1) (Proteintech, Cat# 22952-1-AP), anti-ITGB5 (Proteintech, Cat# 28543-1-AP) and anti-S100A8 (Proteintech, Cat# 15792-1-AP), mouse anti-CD68 (Santa Cruz, Cat# sc-20060) and anti-CD44 (Proteintech, Cat# 60224-1-Ig), followed by washing in PBS. They were incubated with AlexaFluor-555 labeled goat anti-rabbit antibodies or AlexaFluor-488 labeled goat anti-mouse antibodies (ThermoFisher Scientific, Cat# A-32732 or Cat# A-11029) for 1 h, washed again in PBS, and counterstained with 4’,6’-diamidino-2-phenylindole (DAPI) (Abcam, Cat# AB104139) to visualize nuclei. The sections were imaged using a fluorescence or confocal microscope. Fluorescence intensity and distribution were analyzed by ImageJ 1.54i software to evaluate protein expression and localization.

### In vitro cell culture

THP-1 cells, a human monocytic leukemia cell line, were cultured in RPMI-1640 medium supplemented with 10% FBS and 1% penicillin–streptomycin at a density of 1–2 × 10^5^ cells/mL in a humidified incubator at 37 °C with 5% CO_2_. To induce differentiation, cells were treated with 100 nM phorbol 12-myristate 13-acetate (PMA) (MedchemExpress, Cat# HY-18739) dissolved in dimethyl sulfoxide (DMSO) for 48 h. For the s inflammatory model, cells were stimulated with 100 ng/mL SPP1 protein (MedchemExpress Cat# HY-P70499). RAW264.7 murine macrophage cells were cultured in DMEM supplemented with 10% FBS and 1% penicillin-streptomycin. To induce differentiation into macrophages, RAW264.7 cells were treated with 20 ng/mL recombinant murine GM-CSF for 3–7 days, with fresh medium and GM-CSF replenished every 2–3 days.

### Flow cytometry analysis of THP-1 cells

Cells were first stained with Zombie UV viability dye (1:500; BioLegend, Cat# 423108) at room temperature for 20 min. THP-1 cells were washed and Fc-blocked using Human TruStain FcX™ (1:100, 10 min, 4 °C; BioLegend, Cat# 156604). After fixation and permeabilization, the cells were washed and then stained with PE anti-mouse/rat/human MCP-1.

### siRNA-mediated transient genetic inactivation

THP-1 cells were maintained in RPMI-1640 with 10% FBS at 37 °C, 5% CO_2_. On the day of transfection, cells were resuspended in antibiotic-free complete medium; 1–5 × 10^5^ cells were seeded per well in 24-well plates (final volume 500 µL/well). siRNA-lipid complexes were prepared at room temperature using riboFECT™ CP (RiboBio, Cat# C10511-05) following the manufacturer’s instructions to yield 50 nM siRNA (RiboBio, Cat# SIGS0002342-4) in the well; mixtures were incubated 10–15 min before addition to cells. Cells were cultured 48 h post-transfection. Knockdown was assessed by immunoblotting at 48 h. Non-targeting (scramble) siRNA and mock (no siRNA) served as controls. Each condition was performed in technical triplicate and repeated in at least three independent experiments.

### Lentiviral transduction and screening for stable cell lines

The SPP1 knockdown and control lentiviral vectors were constructed by Cyagen Bioscience Co., Ltd. The SPP1-knockdown (*SPP1*-KD), and control (*SPP1*-NC) were generated by lentiviral transduction according to the manufacturer’s protocol. The cells with stable expression were identified by screening in culture medium supplemented with puromycin (Yeasen) at a final concentration of 10 μg/mL after 3 days. The sequences used for knockdown of specifc targets are listed in Table S3.

### Western blotting

For total protein extraction, cells were lysed in RIPA buffer supplemented with a protease inhibitor cocktail. Equal amounts of protein (20 µg per lane) were separated on Bis-Tris-SDS-PAGE gels and transferred to polyvinylidene difluoride (PVDF) membranes. Membranes were blocked with 5% bovine serum albumin (BSA) for 1 h at room temperature and incubated overnight at 4 °C with primary rabbit antibodies against SPP1 and GAPDH (Proteintech, Cat# 10494-1-AP). After washing, membranes were incubated with species-appropriate HRP-conjugated secondary antibodies for 1 h at room temperature. Signals were developed using a 3,3′-diaminobenzidine (DAB) substrate kit and captured on a digital imaging system.

### Transwell assay

For the migration assay, Transwell inserts (Corning; pore size 0.8 μm) were placed in a 24-well plate. The transfected macrophages together with the inducers were added to the lower chambers. A375 cell suspensions were seeded into the upper chambers, and the plate was incubated at 37 °C for 24 h. After migration, the inserts were removed, fixed with 4% paraformaldehyde for 15 min, stained with 0.1% (v/v) crystal violet for 15 min, and imaged.

### Cell proliferation and cell migration assays

To assess cell proliferation, A375 cells were divided into four experimental treatment groups: Control, SPP1, Control + CD44 inhibitor, and SPP1 + CD44 inhibitor. Following the respective treatments, the cells were fixed with 4% paraformaldehyde (PFA) for 15 min at room temperature and processed for Ki67 immunofluorescence (IF) staining. Additionally, cell proliferation and viability were evaluated using a Cell Counting Kit-8 (CCK-8) assay, according to the manufacturer’s instructions.

Cell migration was evaluated using both Transwell and wound healing assays. For the Transwell assay, inserts were placed in a 24-well plate to physically separate the cell populations. THP-1-differentiated macrophages were seeded in the lower chambers, while A375 tumor cells were seeded into the upper chambers. The cells were co-cultured until distinct migration and colony formation were observed.

For the wound-healing assay, A375 cells were seeded into six-well plates one day prior to the experiment. Once the cells reached confluence, the monolayer was artificially wounded by scratching with a sterile 200 µL pipette tip (designated as 0 h). The standard culture medium was discarded, and the cells were washed. Next, the cells were incubated in media corresponding to four distinct treatment groups: Control, SPP1, Control + CD44 inhibitor, and SPP1 + CD44 inhibitor. Representative images of five non-overlapping fields were captured at 0 h and 24 h post-wounding to evaluate the cell migration rate.

### In vivo B16F10 mouse experiment

Subcutaneous Tumor Model and Anti-OPN1 Treatment: Male C57BL/6 mice (8–10 weeks old) were subcutaneously injected with 2 × 10⁵ B16-F10 cells. Five days post-injection, mice were randomly assigned to treatment groups and treated with either an anti-OPN1 antibody (MedChemExpress, Cat# HY-P990116) or an IgG2a isotype control (MedChemExpress, Cat# HY-P990005). Treatments were administered weekly. Tumor growth and mouse body weights were monitored regularly. Tumor volumes were calculated using the standard formula: volume = (length × width²)/2.

To evaluate the tumor-promoting role of SPP1⁺ macrophages in vivo, a tongue co-transplantation model was established using six-week-old male C57BL/6 mice. B16-F10 cells and pretreated, transfected macrophages cultured in 10-cm dishes were washed with pre-chilled PBS and detached using 0.25% trypsin (Gibco) and Accutase, respectively. Cells were collected by centrifugation and resuspended at a B16-F10 tumor cell-to-macrophage ratio of 7:3. The cell mixture was injected into the tongue using insulin syringes. To establish a spontaneous lymph node (LN) metastasis model, cells suspended in 50 μL DMEM were injected submucosally at the border of the tongue. Fourteen days after injection, tumors were harvested, weighed, and photographed. The tissues were subsequently fixed, embedded in paraffin, and subjected to immunohistochemistry (IHC) and multiplex immunofluorescence (mIF) analyses.

### Immunofluorescence analysis of mouse paraffin-embedded tissue specimens

Deparaffinization and antigen retrieval were performed on paraffin-embedded mouse tumor sections as described above for hematoxylin-eosin (H&E) and IF staining. Sections were then blocked with 10% (v/v) normal goat serum to reduce nonspecific/Fc-mediated binding. Primary antibodies were applied and incubated at 4 °C overnight. After five washes with PBST, the sections were incubated with fluorophore-conjugated secondary antibodies at room temperature for 1 h. Nuclei were counterstained with 4′,6-diamidino-2-phenylindole (DAPI) for 15 min.

### Flow cytometry analysis of mouse tissue specimens

Mouse tumor was digested into single-cell suspensions with Liberase™ TL. Cells were first stained with Zombie UV viability dye (1:500) at room temperature for 20 min. The cells were washed and Fc-blocked using TruStain FcX™ PLUS (anti-mouse CD16/32) (1:500, 10 min, 4 °C) (BioLegend, Cat# 156604). The cells were washed and then stained with primary antibodies Brilliant Violet 785 anti-CD45 (BioLegend, Cat# 103149), Brilliant Violet 711™ anti-CD11b (Cat#101241), Brilliant Violet 605™ anti-Ly-6G (Cat# 127639), APC/Cyanine7 anti-Ly-6C (Cat# 128025), FITC anti-F4/80 (Cat# 123107), PE anti-CD206 (Cat# 141706), PE-Cyanine7 anti-CD86 (Cat# 25-0862-82), and PE anti-MCP.1 (Cat# 505903) on ice for 30 min. The samples were detected on a Cytek Aurora. All flow cytometry data were analyzed with Flowjo v10 software (Tree Star).

### Quantification and statistical analysis

Details of statistical analyses are provided in the figure legends and relevant methods subsections. Sample sizes were not predetermined by statistical methods. Data met the assumptions of the tests applied; normality was assessed before conducting parametric analyses. Statistical significance was defined as *P* < 0.05.

## Results

### LN^+^ AM exhibited enhanced myeloid cell infiltration and predominant SPP1

**Fig. 1:**
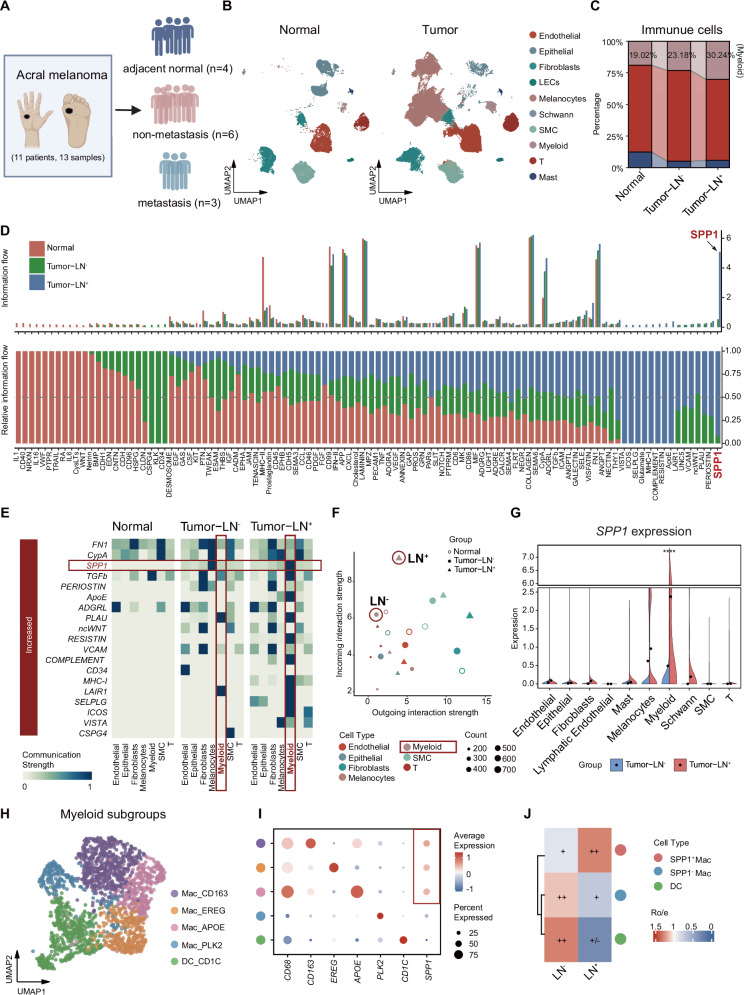
Single-cell transcriptomic landscape reveals cellular heterogeneity and intercellular communication remodeling in acral melanoma (AM) with lymph node metastasis. **A** Schematic representation of the study design. AM patients with (LN^+^) and without lymph node metastasis (LN^-^) were enrolled, and their primary tumor lesions were collected for single cell RNA sequencing (scRNA-seq). **B** UMAP plot demonstrating the cell distribution from primary AM tissues, color-coded by the annotated cell types. LECs, Lymphatic endothelial cells; SMC, smooth muscles cell. **C** Stacked bar chart indicating the fraction of annotated immune cell subtypes originating from patients with adjacent normal, LN^-^ and LN^+^ AM; Percentage of the cell count of the Myeloid cell. **D** CellChat analysis reveals active signaling pathways in three subgroups (Red: Normal, Green: Tumor-LN^-^, Blue: Tumor-LN^+^). **E** Heatmap showing Normal, LN^-^ and LN^+^ AM cell interaction pathways identified according to each cell type. **F** Scatter plot showing outgoing and incoming interaction strength of each cell type in LN^-^ and LN^+^ AM. **G** Split violin plots showing SPP1 expression across annotated cell subclusters. Statistical significance was assessed using an unpaired two-tailed Student’s *t* test; ****P < 0.0001. **H** UMAP plot demonstrating the distribution of myeloid, color-coded by the annotated cell types. **I** Dot plot identifying the expression of marker genes in the annotated myeloid subgroups. **J** Heatmap showing the distribution preference of myeloid subtypes across LN conditions, quantified by the ratio of observed to expected cell counts (Ro/e). Symbol annotations denote Ro/e ranges: +++ (Ro/e > 1.5), ++ (1 < Ro/e ≤ 1.5), + (0.5 ≤ Ro/e ≤ 1), +/− (0 < Ro/e < 0.5), and − (Ro/e = 0).

To gain deeper insights into the acral melanoma (AM) microenvironment, we conducted scRNA-seq on nine tumor samples and four adjacent normal tissue samples from eleven AM patients (Fig. 1A). Integrated cell annotation using classical marker validation resolved 10 transcriptionally distinct populations (Fig. S1A-C). Melanocytes (PMEL^+^/TYRP1^+^/MLANA^+^; 51,663 cells, 43.7%) predominated, accompanied by structural and immune compartments, including endothelial cells, fibroblasts, epithelial lineages, T cells (5924), myeloid cells (2006), and minor stromal/immune subsets.

Stratification of samples into adjacent normal tissues (Normal), non-metastatic lymph node tumors (Tumor-LN^-^), and metastatic lymph node tumors (Tumor-LN^+^) revealed a dynamic reorganization of the tumor microenvironment (Fig. 1B, C). Notably, myeloid cell proportions escalated gradually from 19.02% in normal tissues to 23.18% in Tumor-LN^-^, peaking at 30.24% in Tumor-LN^+^, linking lymph node metastasis with increased myeloid infiltration (Fig. 1C).

To investigate the dynamics of cell-cell communication linkages during AM progression, we utilized CellChat, identifying 106 significant signaling pathways (Fig. 1D). Among the 19 pathways differentially activated in tumor samples, SPP1 signaling demonstrated striking tumor-specific enrichment, featuring an 11.5-fold increase in interaction strength in Tumor-LN^+^ versus Tumor-LN^-^ groups (Fig. 1E). Myeloid cells emerged as the primary mediators of both outgoing and incoming SPP1 interactions, particularly in Tumor-LN^+^ samples (Fig. 1F). Consistent with patient cohort findings, SPP1 expression was significantly elevated in Tumor-LN^+^ tissues, with the most pronounced upregulation in the myeloid compartment (Fig. 1G).

Building on these findings regarding myeloid cells’ central role in SPP1 signaling, we conducted unsupervised clustering of 2,006 myeloid cells based on their transcriptional profiles, yielding five distinct clusters: CD163^+^ macrophages (M2-like and immunoregulatory) [31]; EREG^+^ macrophages (epiregulin-expressing, pro-inflammatory, and tissue-remodeling); APOE^+^ macrophages (apolipoprotein E-associated and lipid metabolism-related); PLK2^+^ macrophages (polo-like kinase 2-associated and stress-responsive) [32]; and CD1C^+^ dendritic cells (classical type 2 DCs involved in antigen presentation). These clusters underscore the functional heterogeneity within the myeloid compartment of the tumor microenvironment (Fig. 1H). Furthermore, the corresponding dot plot displays the marker genes for cell annotation, validated through manual inspection of gene expression patterns across the clusters. Notably, SPP1 expression was predominantly confined to macrophage subsets, showing elevated levels in CD163^+^, EREG^+^, and APOE^+^ macrophages but low levels in PLK2^+^ macrophages (Fig. 1I). Accordingly, we classified CD163^+^, EREG^+^, and APOE^+^ macrophages as SPP1^+^ macrophages, while designating PLK2^+^ macrophages as SPP1^-^ macrophages. Ro/e analysis subsequently revealed preferential enrichment of SPP1^+^ macrophages in LN^+^ tumors, in contrast to the relative enrichment of SPP1^-^ macrophages and CD1C^+^ DCs in LN^-^ tumors (Fig. 1J).

### SPP1^+^ macrophages correlate with poor prognosis and reinforce M2-related TME

**Fig. 2:**
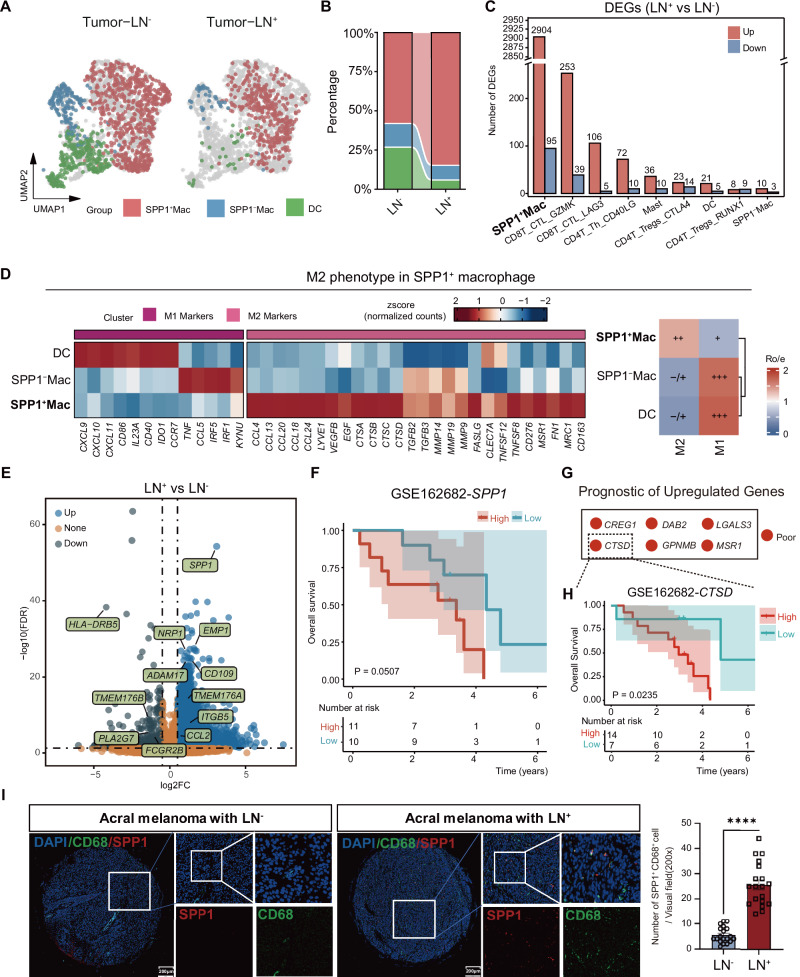
SPP1^+^ macrophages are enriched in LN^+^ acral melanoma (AM) and associate with M2 polarization and poor clinical outcomes. **A** UMAP plot illustrating the distribution of myeloid in Tumor- LN^-^ and Tumor-LN^+^. **B** Stacked bar chart showing the composition of myeloid cell types in Tumor- LN^-^ and Tumor-LN^+^. **C** Bar plot displaying the total count of upregulated and downregulated genes within each major immune cell type when comparing LN^+^ to LN^-^ samples. **D** Heatmap displaying the relative expression levels of M1 (pro-inflammatory) and M2 (anti-inflammatory) signature genes in different myeloid clusters (left). The heatmap showing the macrophage polarization preference of myeloid subtypes, as determined by the ratio of observed to expected cell counts (Ro/e) (right). Ro/e value ranges: +++ (Ro/e > 1.5), ++ (1 < Ro/e ≤ 1.5), + (0.5 ≤ Ro/e ≤ 1), +/− (0 < Ro/e < 0.5), and − (Ro/e = 0). **E** Volcano plot of differentially expressed genes in SPP1^+^ macrophages from LN^+^ versus LN^-^ AM. **F** Kaplan-Meier survival curves comparing overall survival (OS) in AM patients (GSE162682 cohort, *n* = 21) stratified by optimal SPP1 mRNA expression cutoff. **G** Clinical prognostic value of SPP1^+^ macrophage subtype in AM patients, stratified by gene expression-based optimal cutoff values. Red: high expression associated with poor survival. **H** Prognostic evaluation of CTSD, the top-ranked differentially expressed gene among candidates associated with poor prognosis in (**G**). **I** Representative immunofluorescence (IF) staining image of SPP1 and CD68 macrophage cells in LN^-^ and LN^+^. Scale bars= 200 μm. Histogram illustrating the quantification of the percentage of SPP1-positive cells in LN^-^ and LN^+^ tissues. Data presented as mean ± SEM, **** *P* < 0.0001 (unpaired student’s *t* test).

We next delineated macrophage subsets based on SPP1 expression and compared their distribution across Tumor-LN^-^, and Tumor-LN^+^ groups (Fig. 2A). This analysis revealed a significant enrichment of SPP1^+^ macrophages in LN^+^ AM (86.43%) compared to LN^-^ AM (60.78%) (Fig. 2B). Across immune compartments, SPP1^+^ macrophages exhibited prominent expression of differentiation genes, indicating they undergo significant changes (Fig. 2C).

To further characterize their functional state, we performed signature scoring for macrophage polarization demonstrated that SPP1^+^ macrophages consistently exhibited higher M2-like scores, supporting an M2-skewed trajectory during progression (Fig. 2D), a finding validated by an external public scRNA-seq dataset (Fig. S2A-D). We then conducted gene set enrichment analysis (GSEA)-based pathway enrichment analysis comparing SPP1^+^ macrophages (SPP1^+^ Mac), SPP1- macrophages (SPP1^-^ Mac), and DC populations (Fig. S2E). This revealed significant enrichment of canonical M2-associated functional programs in the upregulated gene sets of SPP1⁺ macrophages, including immunosuppression (complement receptor signaling, TGF-β pathway, apoptotic cell clearance, phagocytosis) [33], invasion and ECM remodeling (EMT, MMP activation, ECM degradation, collagen catabolism, angiogenesis) [34], and lipid metabolism pathways—all hallmarks of M2-like protumor macrophage polarization [35]. In contrast, APC activation pathways (antigen presentation, T cell activation, IFN-γ signaling, IL-12 production) were significantly enriched in the downregulated gene sets of SPP1⁺ macrophages, consistent with their immunosuppressive rather than immunostimulatory phenotype. These findings provide transcriptomic evidence substantiating the M2-like functional identity of SPP1⁺ macrophages within the LN⁺ tumor microenvironment.

Building on this, differential expression analysis comparing SPP1^+^ macrophages in LN^+^ versus LN^-^ tumors identified upregulation of SPP1-associated receptor components, such as the integrin subunit beta (ITGB5), along with elevated expression of the regulatory monocyte chemokine gene CCL2 in LN^+^ tumors (Fig. 2E). Additionally, LN^+^ SPP1^+^ macrophages upregulated genes implicated in pro-metastatic crosstalk and immunosuppressive reprogramming (e.g., NRP1 [36], ADAM17 [37], CD109 [38], EMP1 [39]), whereas LN^-^ SPP1^+^ macrophages retained a relatively antigen-presenting phenotype, highlighted by increased MHC-II (HLA-DRB5) and antigen-processing–associated genes (TMEM176B [40, 41], PLA2G7 [42], FCGR2B [43]) (Fig. 2E).

Complementing our single-cell analysis, we systematically evaluated the clinical relevance of SPP1 to validate SPP1^+^ macrophages as central mediators of AM progression. Specifically, Kaplan-Meier analysis confirmed SPP1 over-expression as an independent prognostic marker for reduced survival (Fig. 2F). This finding was further substantiated by multivariate survival analysis of six SPP1^+^ macrophage-specific genes (*CREG1, CSTD, DAB2, GPNMB, LGALS3, MSR1*), derived from our single-cell profiling, which showed consistent correlation with decreased overall survival (Fig. 2G, H and Fig. S4). Notably, cathepsin D (*CTSD*) emerged among the top three genes with the strongest prognostic contribution within this signature (Fig. 2H), with detailed results for the other components shown in Fig. S4. As reported in the literature, cathepsin D has been shown to actively drive M2-like TAM polarization and promote metastatic progression via the TGFBI–CCL20 signaling axis, supporting a more malignant interpretation of the SPP1^+^ macrophage-associated signature [44].

To validate these results, we performed immunofluorescence analysis on tissue microarrays from 25 AM patients. Quantification revealed that SPP1 was predominantly expressed in CD68^+^ macrophages, with minimal levels in LN^-^ specimens compared to marked elevation in LN^+^ metastasis (Fig. 2I). Together, these results collectively underscore the critical role of SPP1^+^ macrophages in AM progression and their potential as a prognostic indicator.

### Enhanced SPP1 autocrine signaling in LN^+^ macrophages induces ITGB5-mediated CCL2 upregulation

**Fig. 3:**
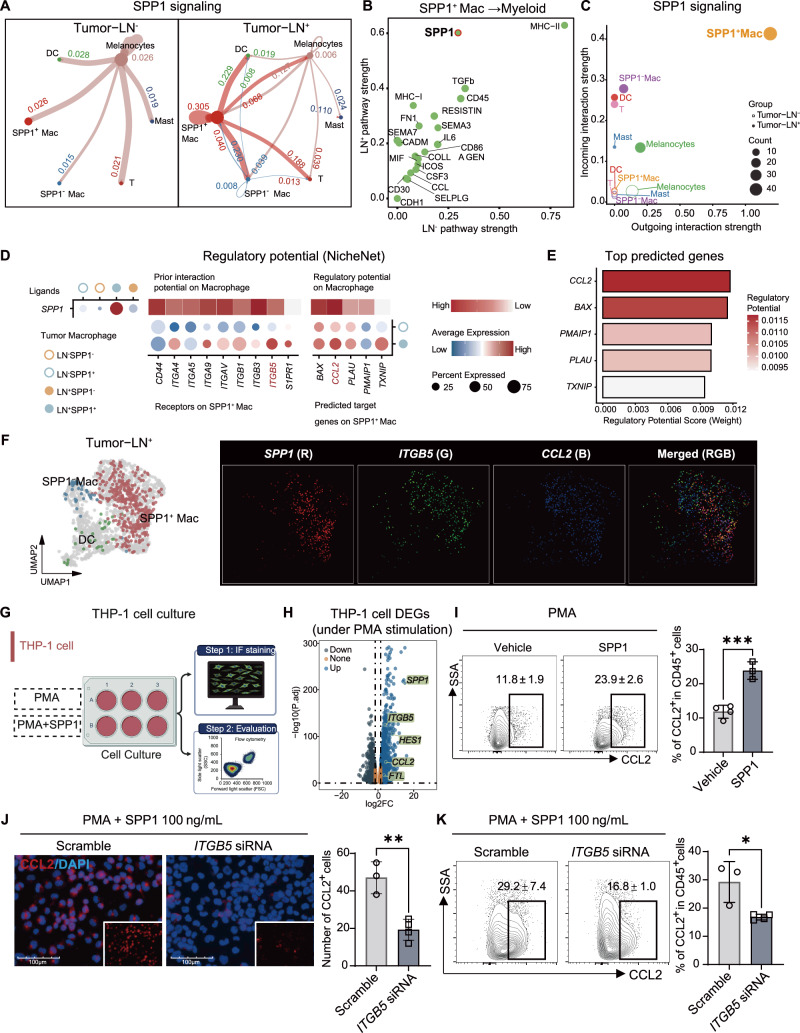
Autocrine SPP1 signaling in LN^+^ macrophages induce ITGB5-mediated CCL2 upregulation. **A** Circle plot depicting intercellular communication via the SPP1 signaling pathway in Tumor-LN^-^ and Tumor-LN^+^ datasets. Node color indicates signaling source, and edge line thickness reflects inferred interaction strength. Node size represents the number of cells per group. Numbers indicate intercellular interaction strength, with the color matching the target cell. **B** Scatter plot showing the outgoing and incoming interaction strength between SPP1^+^ macrophages and myeloid cells in LN^−^ and LN^+^ AM. **C** Scatter plot showing the outgoing and incoming interaction strength of individual cell types within the SPP1 signaling pathway, as inferred by CellChat. Each point represents a cell type, with color indicating cell identity and point size reflecting the total number of SPP1-associated interactions. Filled circles correspond to LN^+^ tumors, and open circles correspond to LN^-^ tumors. **D** NicheNet-based ligand–receptor–target analysis. Dot plots indicating the expression of ligands (left), receptors (middle, bottom), and predicted target genes (right, bottom) in SPP1^+^ macrophages across the indicated tumor macrophage subsets. The heatmaps above summarize the prior interaction potential and regulatory potential calculated by NicheNet. **E** Bar plot showing the ranked target genes in SPP1^+^ macrophages predicted by NicheNet analysis. Genes are ordered on the y-axis based on their regulatory potential scores. The length of the bars and the color gradient (from white to dark red) correspond to the regulatory potential weight, with higher values indicating a higher probability of being regulated by SPP1 in an autocrine manner. **F** UMAP projection of myeloid cells delineating three major clusters (left). Fluorescence-like single-cell feature maps (right) generated by encoding normalized expression of SPP1, ITGB5, and CCL2 into the red (R), green (G), and blue (B) channels, respectively, together with an RGB composite that approximates multiplex immunofluorescence. The merged image facilitates visualization of cell-level co-expression patterns and spatial concordance of these genes at single-cell resolution. **G** Schematic illustrating the experimental workflow for validating SPP1 autocrine signaling in myeloid cells, including PMA-induced differentiation of THP-1 cells followed by immunofluorescence and flow cytometry analysis. **H** Volcano plot of RNA-seq data from THP-1 monocytes stimulated with 100 ng/mL phorbol 12-myristate 13-acetate (PMA) for 24 h vs. untreated controls. **I** Flow cytometry analysis and histogram quantification between vehicle and SPP1-stimulated cells. Data presented as mean ± SEM, ***P < 0.001 (unpaired student’s *t* test). **J** Representative immunofluorescence images (left) of CCL2 and quantification of the number of CCL2^+^ cells per field (right). Data are presented as mean ± SEM, ** *P* < 0.01 (unpaired student’s *t* test). Scale bar = 100 μm**. K** Flow cytometry analysis of CCL2 expression and quantification of the percentage of CCL2^+^ cells. Data are presented as mean ± SEM, * *P* < 0.05 (unpaired student’s *t* test).

To further characterize the prominent effect of SPP1 in TME across LN^-^ and LN^+^, we used CellChat to systematically analyze the SPP1 signaling pathway (Fig. 3A–C). This analysis identified SPP1^+^ macrophages as the dominant signaling hub and revealed a strong autocrine SPP1 circuit within this subset. Building on these findings, we integrated NicheNet ligand–receptor inference with differential expression analysis to resolve downstream regulatory programs. In the LN^+^ group, the SPP1 receptor ITGB5 and the key monocyte chemoattractant CCL2 were both significantly upregulated (Fig. 3D). Consistently, NicheNet weighted network analysis ranked CCL2 among the top high-weight target genes downstream of SPP1 (Fig. 3E), suggesting that SPP1 may promote CCL2-associated monocyte recruitment via ITGB5-mediated signal transduction. At the single-cell level, feature plots on the scRNA-seq UMAP demonstrated highly concordant expression patterns of SPP1, ITGB5, and CCL2 (Fig. 3F). Notably, all three genes showed pronounced and overlapping enrichment within the SPP1^+^ macrophage subset, and the merged visualization further supported their strong co-expression.

To validate the functional significance of SPP1 in a controlled setting, we utilized a THP-1 monocyte model differentiated into macrophages following 24-hour PMA treatment [45](Fig. 3G, H). Flow cytometric analysis (Fig. 3I) demonstrated that treatment with exogenous SPP1 (100 ng/mL) significantly increased CCL2 production in differentiated macrophages. To define the essential role of ITGB5 in SPP1-driven CCL2 induction, ITGB5 was knocked down in macrophages prior to SPP1 stimulation. Immunofluorescence analysis demonstrated that ITGB5 depletion markedly reduced the number of CCL2-positive cells induced by SPP1 (Fig. 3J). This effect was further confirmed by flow cytometry, which revealed a pronounced decrease in the proportion of CCL2^+^ cells in the ITGB5 knockdown group compared with the corresponding control (Fig. 3K). Together, these protein-level data support ITGB5 as a critical receptor required for SPP1-mediated upregulation of CCL2.

Collectively, intercellular communication mediated by SPP1^+^ macrophages is substantially enhanced in the LN^+^ metastatic immune microenvironment. Activation of the SPP1–ITGB5 axis drives downstream CCL2 expression, which may promote recruitment of additional monocytes/macrophages and thereby contribute to the establishment of a metastasis-associated immunosuppressive niche.

### SPP1-CD44 axis promotes S100A8^+^ melanoma subsets in LN^+^ TME

**Fig. 4:**
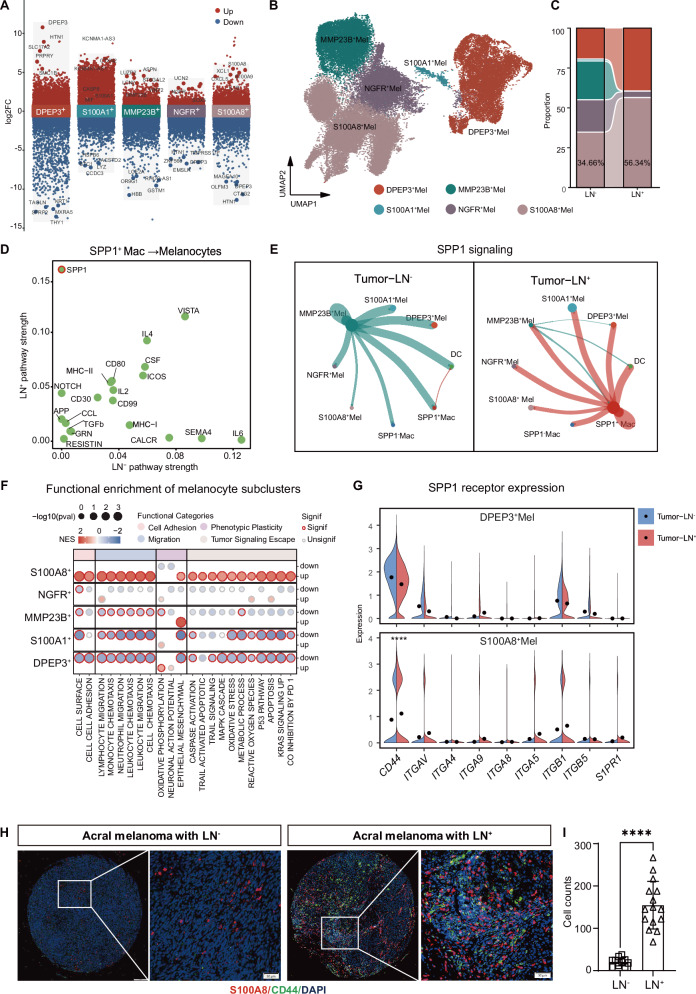
S100A8^+^ melanocyte subpopulation is enriched in LN^+^ acral melanoma and associated with SPP1-mediated intercellular communication. **A** Volcano plot showing differentially expressed genes (DEGs) across melanocyte subclusters. **B** UMAP plot showing the cell distribution from melanocytes, color-coded by the annotated subtypes. **C** River plot illustrating differences in cell subpopulation composition between the Tumor- LN^-^ and Tumor-LN^+^ groups. **D** Scatter plot showing the outgoing and incoming interaction strength between SPP1^+^ macrophages and melanocytes in LN^−^ and LN^+^ AM. **E** Circle plot depicting intercellular communication via the SPP1 signaling pathway in Tumor-LN^-^ and Tumor-LN^+^ datasets. Node color indicates signaling source, and edge line thickness reflects inferred interaction strength. Node size represents the number of cells per group. **F** Pathway enrichment analysis across five melanoma cell subclusters. Selected Hallmark, GO, and Reactome pathways were analyzed using a multi-group enrichment approach. **G** Faceted violin plots showing SPP1 receptor expression (CD44 and integrin subunits) in Tumor–LN^−^ (blue) versus Tumor–LN^+^ (red) samples, focusing on the two melanocyte subclusters with the highest LN^+^ representation (DPEP3^+^Mel and S100A8^+^Mel). **H** Representative immunofluorescence (IF) staining image of S100A8 and CD44 melanocytes in LN^−^ and LN^+^. Scale bars = 50 μm. **I** Quantification of the percentage of S100A8^+^ CD44^+^ cells in LN^-^ and LN^+^ tissues. Data are presented as mean ± SEM. Statistical significance was determined by unpaired student’s *t* test. ****P < 0.0001.

Intriguingly, SPP1^+^ macrophages not only modulate immune responses but also directly regulate melanoma cell plasticity. We comprehensively analyzed melanocyte heterogeneity and identified five principal clusters by unsupervised clustering (resolution = 0.3). These clusters resolved into distinct neoplastic subpopulations (Fig. 4A, B). Systematic characterization revealed five molecular subsets: (1) DPEP3^+^ clusters with an epigenetically activated HTN1/SMC1B signature; (2) proliferative S100A1^+^ niches enriched in cell-cycle regulators CASP8 and MIF; (3) metabolic MMP23B^+^ pools showing LCN2 and WNT2 overexpression; (4) NGFR^+^ subsets marked by SOX5 co-expression and perineural invasion potential; and (5) inflammatory S100A8^+^ populations with IL1B/CXCL5-driven myeloid recruitment signatures (Fig. 4A).

Applying these clustering criteria to LN^-^ and LN^+^ samples, we found that LN^-^ specimens exhibited a balanced subpopulation distribution, whereas LN^+^ cases showed pronounced expansion of DPEP3^+^ (39.53%) and S100A8^+^ (56.34%) malignant clusters (*χ*² = 47.9, *P* = 1.012e-09; Fig. 4C). Furthermore, CellChat comparative mapping revealed a striking shift in the tumor microenvironment: SPP1 signaling was predominantly melanocyte-driven in LN^-^ regions, but transitioned to a SPP1^+^ macrophage-orchestrated pattern with higher interaction weight in LN^+^ regions (Fig. 4D, E).

To identify the tumor cell subsets most responsive to SPP1 signaling, we performed GSEA across the five malignant melanocyte subpopulations to functionally annotate their biological programs. The S100A8^+^ subset displayed significant enrichment of pathways related to cell adhesion and cell migration, accompanied by activation of programs associated with tumor immune evasion (Fig. 4F). This enrichment profile suggests that S100A8^+^ malignant cells may possess heightened motility and immune-adaptive capacity, making them more likely to expand or undergo functional remodeling under metastasis-associated selective pressures, and potentially representing a key responder population to myeloid-derived cues. Given that LN^+^ tumors showed a marked expansion of the S100A8^+^ and DPEP3^+^ malignant clusters (Fig. 4C), we prioritized these two enriched subsets as putative SPP1-responsive populations. We therefore compared the expression of multiple candidate SPP1 receptors across S100A8^+^ and DPEP3^+^ tumor cells between LN^−^ and LN^+^ samples to identify the receptor most likely mediating SPP1 effects in the LN^+^ microenvironment. Notably, CD44 was selectively and significantly upregulated in LN^+^ S100A8^+^ tumor cells, whereas other candidate receptors were either expressed at low levels overall or exhibited LN-associated trends inconsistent with metastatic progression (Fig. 4G). These results nominate CD44 as the most plausible receptor amplifying SPP1 signaling in LN^+^ tumors and support an SPP1–CD44 axis through which SPP1^+^ macrophages may preferentially influence the S100A8^+^ melanoma state.

Building on the SPP1–CD44 axis, we used the KEGG CD44 pathway as a scaffold to overlay LN^+^ versus LN^−^ differential expression in S100A8^+^ tumor cells (Fig. S5). LN^+^ S100A8^+^ cells showed coordinated activation of CD44v3-consistent modules, including LARG–PLCs–IP3R–Ca^2+^–CaMKII [46], NHE-1–Gab1–PI3K–AKT [47, 48], and Ankyrin–Tiam1–Rac1–PAK1 [49] branches, supporting enhanced cytoskeletal remodeling, survival signaling, and directional migration.

To further validate these observations at the protein level, we performed immunofluorescence staining on tissue microarrays, revealing a higher abundance of S100A8^+^ CD44^+^ cells in the LN^+^ group (Fig. 4H, I). These results underscore the pivotal role of SPP1-CD44 signaling in promoting lymph node metastasis.

### SPP1^+^ macrophages induced S100A8 expression and enhanced melanoma malignancy in vitro

**Fig. 5:**
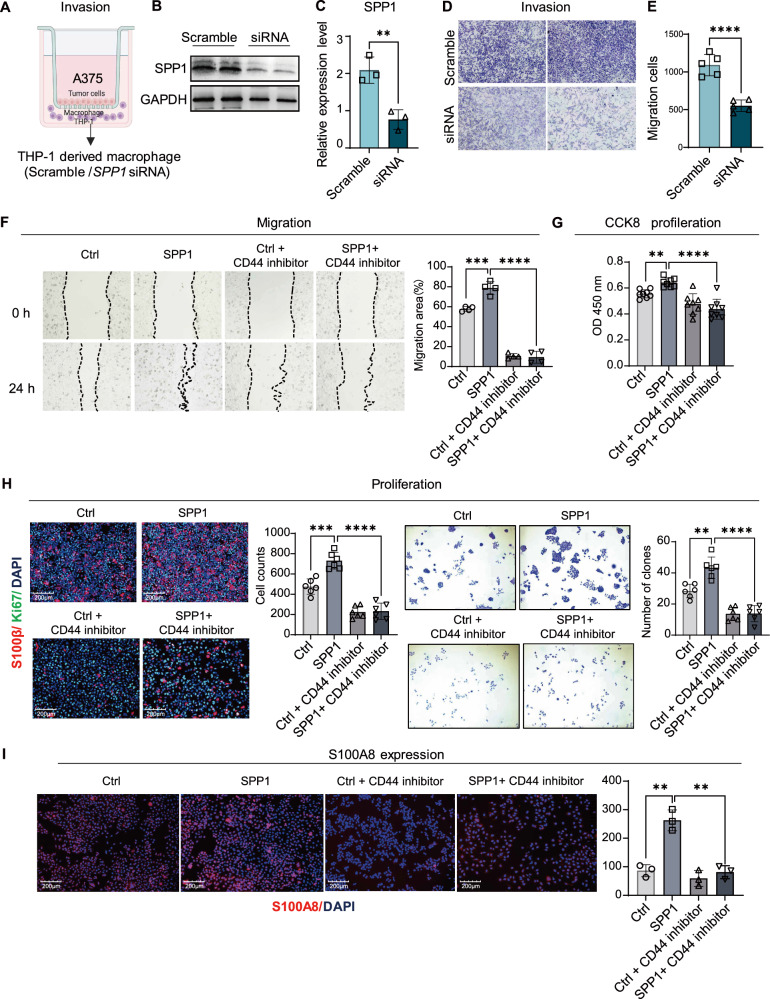
SPP1 enhances melanoma cell migration, proliferation, and S100A8 expression via CD44 signaling. **A** Schematic overview of the in vitro models for macrophage-tumor cell interaction. **B** Representative image showing SPP1 protein levels; GAPDH was used as a loading control. **C** Quantification of SPP1 protein expression normalized to GAPDH in each treatment group. Data are presented as relative expression levels (mean ± SEM, *n* = 3). **P < 0.01 (unpaired Student’s *t* test). **D** Transwell migration assay of A375 cells following SPP1 knockdown by siRNA. **E** Quantification of migrated cell numbers from five independent experiments (*n* = 5). Data are presented as mean ± SEM. ****P < 0.0001 (unpaired student’s *t* test). **F** Representative wound healing images at 0 h and 24 h under the indicated treatments (Ctrl, SPP1, Ctrl + CD44 inhibitor, and SPP1 + CD44 inhibitor). Dashed lines indicate wound boundaries. **G** CCK-8 assay assessing cell proliferation under the indicated treatments. **H** Representative immunofluorescence staining of S100β and Ki67 in melanoma cells under the indicated treatments (left). Scale bars = 200 μm. Quantification of Ki67-positive cells is shown. Representative colony formation images and quantification of colony numbers (right). **I** Representative immunofluorescence staining and quantification of S100A8 in melanoma cells. Scale bars = 200 μm. Data presented as mean ± SEM, *n* = 3, **P < 0.01 (unpaired student’s *t* test).

Given prior evidence linking SPP1 to enhanced tumor cell migration, we employed transwell chambers for invasion and migration assays: tumor cells (A375) were placed in the upper chamber, while THP-1-derived macrophages (with or without siRNA) were in the lower chamber, both under SPP1 stimulation to mimic the LN^+^ environment (Fig. 5A). we used siRNA to downregulate SPP1 in THP-1 cells, which were induced to differentiate into macrophages and validated by western blotting (Fig. 5B, C). A375 cells exhibited significantly greater migratory capacity in the control group than in the SPP1-knockdown group (Fig. 5D), as evidenced by a marked reduction in the number of migrated cells following SPP1 downregulation (Fig. 5E).

As CD44 is a major functional receptor for SPP1, so receptor blockade experiments were performed to test whether the pro-migratory phenotype relied on the SPP1–CD44 axis. In scratch assays, exogenous SPP1 accelerated wound closure in A375 cells, whereas CD44 inhibition reduced basal migration and markedly attenuated the SPP1-induced increase (Fig. 5F). Similarly, CCK-8 assays showed increased cell viability after SPP1 treatment, and this increase was significantly blunted by CD44 inhibition (Fig. 5G). Concordantly, Ki67 immunofluorescence revealed a higher fraction of Ki67-positive cells in the SPP1-treated group, which was suppressed upon CD44 blockade. Colony formation assays further indicated that SPP1 enhanced clonogenic capacity, and this effect was abolished by CD44 inhibition (Fig. 5H). Together, these results indicate that SPP1-associated migratory and proliferative phenotypes in A375 melanoma cells are CD44 dependent.

Building on prior bioinformatic findings implicating S100A8 in metastatic processes, S100A8 expression was examined following SPP1 stimulation and CD44 blockade. Immunofluorescence staining demonstrated a robust increase in S100A8 signal intensity in A375 cells upon exogenous SPP1 treatment (Fig. 5I). In contrast, co-treatment with a CD44 inhibitor markedly reduced S100A8 fluorescence and effectively suppressed the SPP1-induced increase.

Collectively, the in vitro macrophage–melanoma co-culture model showed that macrophage-derived SPP1 was associated with enhanced melanoma cell migration and proliferative phenotypes and induced upregulation of the pro-metastatic marker S100A8, and these effects were substantially diminished by pharmacological inhibition of CD44.

### Anti-OPN1 treatment alleviates melanoma progression in vivo and remodels the M2 macrophage microenvironment

**Fig. 6:**
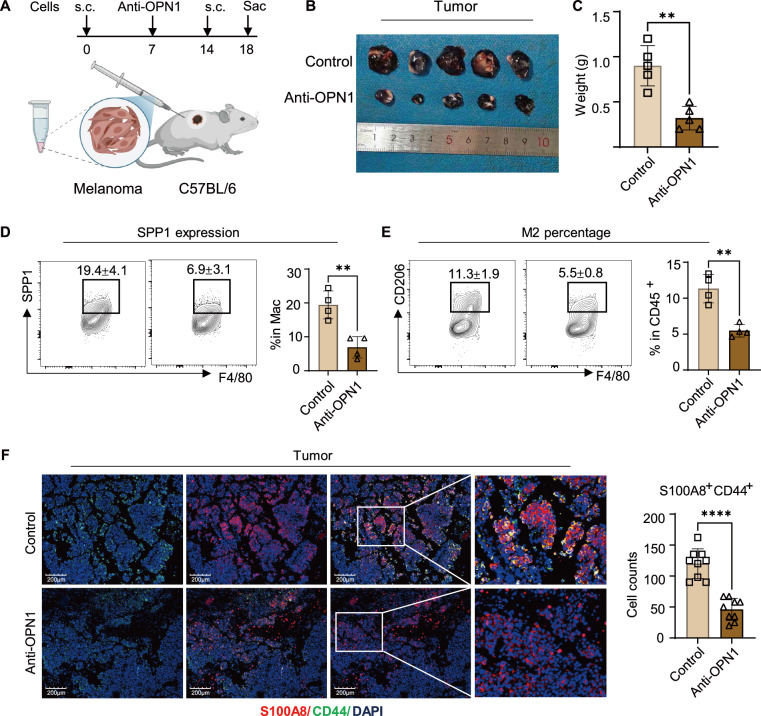
Therapeutic blockade of SPP1 suppresses melanoma growth and remodels the tumor microenvironment. **A** Schematic showing the process for establishing the subcutaneous tumor models and treatment schedule. Tumors were harvested 18 days after cells injection. s.c., subcutaneous. **B** Images of isolated tumors in control and anti-Osteopontin (OPN) 1 group (2.25 mg/kg). **C** Final tumor weights at day 18 in control and anti-OPN1-treated groups. Data are presented as mean ± SEM (*n* = 5 mice per group). **P < 0.01 (unpaired Student’s *t* test). **D** Representative flow cytometry contour plots and quantification of SPP1^+^ cells in macrophages. Data are presented as mean ± SEM, *n* = 4. **P < 0.01 (unpaired Student’s *t* test). **E** Representative flow cytometry contour plots and quantification of the percentage of CD206^+^ cells in CD45^+^ cells from Control and anti-OPN1-treated groups. Data are presented as mean ± SEM, *n* = 4. **P < 0.01 (unpaired Student’s *t* test). **F** Representative IF staining and quantification of S100A8^+^CD44^+^ in tumor cells. Scale bars = 200 μm. Data presented as mean ± SEM, ****P < 0.0001 (unpaired student’s *t* test).

To further validate the in vivo effects of SPP1 in the TME, we administered intra-tumoral injections of anti-osteopontin (OPN)1 (2.25 mg/kg). For sustained efficacy, injections began 1 week after subcutaneous implantation of B16-F10 cells and continued weekly around the tumor site (Fig. 6A). Tumor volumes and weights were significantly reduced in the anti-OPN1 group compared to controls (Fig. 6B, C). Following tumor harvest, we conducted flow cytometry, IF, and HE staining to assess M2 macrophage subtypes, S100A8, and CD44 expression. Flow cytometry further showed that the proportion of SPP1^+^ cells among tumor-associated macrophages was markedly decreased in the anti-OPN1 group compared with controls (Fig. 6D), indicating effective suppression of local OPN/SPP1-associated self-amplifying monocyte self-recruitment signaling. Consistently, flow cytometry revealed a marked decrease in the M2 phenotype, indicating remodeling of the M2-dominant microenvironment and consequent alleviation of tumor burden (Fig. 6E). Additionally, IF analyses showed reduced S100A8 and CD44 expression after anti-OPN1 treatment, collectively demonstrating its potential to inhibit tumor progression (Fig. 6E, F).

### Macrophage-derived SPP1 promotes primary tumor growth and sentinel lymph node metastasis

**Fig. 7:**
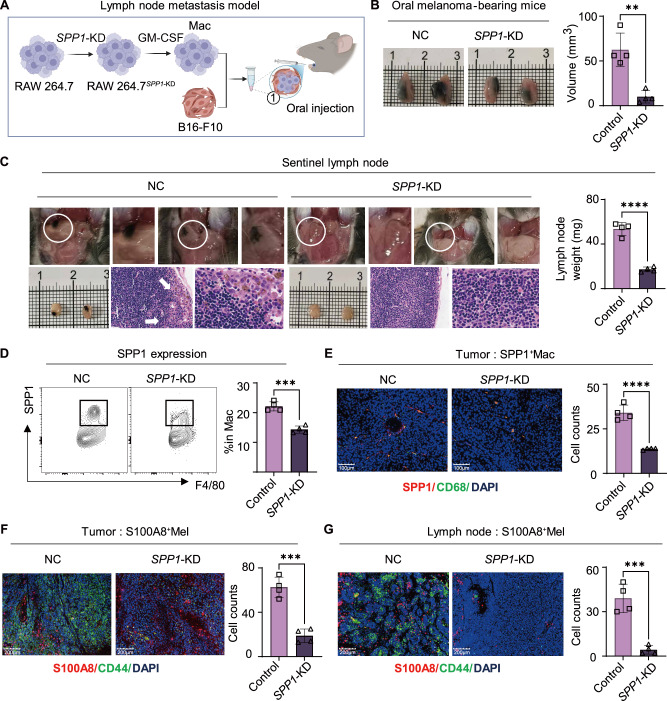
SPP1 knockdown reduces sentinel lymph node metastasis and reshapes the tumor microenvironment in a murine melanoma model. **A** RAW264.7 cells were subjected to SPP1 knockdown (*SPP1*-KD) and stimulated with GM-CSF to generate macrophages. Macrophages were mixed with B16-F10 cells and injected into the tongue of mice to establish the lymph node metastasis model. **B** Tumor volume measured 14 days after injection of B16-F10 cells mixed with NC or *SPP1*-KD macrophages. **C** Representative images include in situ photographs (white circles indicate metastatic lymph nodes), harvested SLNs, and H&E-stained sections (arrows indicate metastatic lesions). Quantification of SLN weights for the NC and *SPP1*-KD groups is shown on the right. Data presented as mean ± SEM, *n* = 4, **** *P* < 0.0001 (unpaired student’s *t* test). **D** Flow cytometric analysis of SPP1 expression and quantification of the percentage of SPP1^+^ cells in the NC and *SPP1*-KD groups. Data presented as mean ± SEM, *n* = 4, ***P < 0.001 (unpaired student’s *t* test). **E** Representative immunofluorescence images and quantification of SPP1^+^ macrophage. Scale bar = 100 μm. Data presented as mean ± SEM, *n* = 4, **** *P* < 0.0001 (unpaired student’s *t* test). **F** Representative immunofluorescence images and quantification of S100A8^+^ CD44^+^ melanoma cells in tumor lesion. Scale bar = 200 μm. Data presented as mean ± SEM, *n* = 4, ***P < 0.001 (unpaired student’s *t* test). **G** Representative immunofluorescence images and quantification of S100A8^+^ CD44^+^ melanoma cells in lymph node lesion. Scale bar = 200 μm. Data presented as mean ± SEM, *n* = 4, *** *P* < 0.001 (unpaired student’s *t* test).

We next investigated whether macrophage-derived SPP1 contributes to melanoma progression and lymph node dissemination. RAW264.7 macrophages were engineered to knock down SPP1 (SPP1-KD) or serve as negative controls (NC), stimulated with GM-CSF, and mixed with B16-F10 cells before orthotopic injection into the oral to establish a sentinel lymph node (SLN) metastasis model (Fig. 7A). 10–14 days after tumor establishment, the SPP1-KD group displayed a significantly reduced primary tumor volume compared with the NC group (Fig. 7B). Examination of SLNs further revealed decreased metastatic incidence and fewer gross metastatic features, and H&E staining confirmed a marked reduction in metastatic foci. In parallel, SLN weight was significantly decreased in the SPP1-KD group (Fig. 7C).

Consistent with effective knockdown, flow cytometry showed a significant decrease in SPP1^+^ cells in the SPP1-KD group (Fig. 7D), and IF staining confirmed reduced intratumoral SPP1^+^CD68^+^ macrophages (Fig. 7E). Importantly, melanoma cells exhibited coordinated suppression of invasion/metastasis-associated phenotypes, with fewer S100A8^+^CD44^+^ cells in primary tumors (Fig. 7F) and metastatic lymph node lesions (Fig. 7G). These findings suggest that targeting SPP1/OPN signaling reshapes the macrophage-driven niche and dampens the S100A8–CD44 pro-metastatic program, thereby restricting metastatic dissemination.

## Discussion

Advanced AM displays a heightened propensity for LN metastasis compared to cutaneous melanoma, contributing to therapeutic resistance and poor clinical outcomes [50]. To investigate this, we conducted scRNA-seq on AM samples with or without LN metastasis, uncovering a distinct SPP1^+^ signaling pathway. Our analysis revealed strong autocrine and paracrine heterogeneity in SPP1 expression within the LN^+^ TME relative to LN^-^. SPP1, also known as osteopontin, is a glycosylated protein with key biological functions including cell adhesion, migration, regulation of innate and adaptive immune responses, cell differentiation, cytokine production, and cancer progression [31, 51]. While SPP1 can be secreted directly by neoplastic cells to drive intrinsic malignant phenotypes, myeloid-derived SPP1 is increasingly recognized as a master regulator of TME remodeling. Recent studies have established a strong correlation between elevated SPP1 levels and various malignancies [52]. Among these, SPP1^+^ macrophages, characterized by high SPP1 expression, have garnered significant attention for their ability to establish physical and immunosuppressive barriers against T-cell infiltration [53].

In line with these observations, our dataset highlighted a prominent SPP1^+^ macrophage subcluster enriched in LN^+^ versus LN^-^ tumors. Interestingly, these cells also exhibited profound expression of differentiation-related and immunomodulatory genes among other immune cell populations, underscoring their high variability and critical importance in the dynamic LN^+^ TME. These results indicated that SPP1^+^ macrophages play a pivotal role in LN^+^ AM. Given the heterogeneity of TME [54], SPP1^+^ macrophages exhibited varying marker profiles across studies, suggesting both conserved and context-dependent functions. Our analysis revealed elevated SPP1 expression in CD163^+^, EREG^+^, and APOE^+^ macrophage subclusters, which are typically aligned with an M2 phenotype. Notably, SPP1 expression is not confined to a single cluster but spanned, potentially reflecting nuanced states within the protumor spectrum—such as the unique transitioned remodeling in our data, which compensated with the broader lipid- and hypoxia-focused profiles in prior studies [55, 56]. Additionally, unlike studies highlighting SPP1^+^ polarity opposite to CXCL9^+^ antitumor macrophages [57], our clusters lack a direct CXCL9-dominant counterpart, suggesting context-specific heterogeneity in the tumor microenvironment that may influence prognosis and therapeutic targeting differently. These findings re-verified through analysis of another public AM dataset. Furthermore, signaling pathways analysis demonstrated prominent autocrine and paracrine interactions between SPP1^+^ macrophages and melanoma subsets in LN^+^. Overall, in LN^+^ melanoma, SPP1^+^ macrophages exhibit a bias toward the M2 phenotype to drive metastasis, featuring unique autocrine and paracrine signaling.

To enhance clinical relevance, previous studies have demonstrated that total intratumoral SPP1 expression correlates with more aggressive tumor behavior, therapy resistance, and reduced overall survival in glioma, breast cancer, colorectal cancer, liver cancer, and other malignancies [58–63]. Despite these insights, the precise role of SPP1^+^ macrophages and their prognostic significance in AM—particularly their specific contributions to LN metastasis—remains largely unexplored. Our analysis demonstrated that high SPP1 expression was associated with reduced survival rates. Furthermore, by specifically selecting genes upregulated in SPP1^+^ macrophages, we reinforced the association with poor prognosis in LN^+^ AM patients.

Numerous studies have shown that SPP1 drives immunosuppression and metastasis across various cancers through diverse mechanisms. Extracellular SPP1 classically exerts its pro-metastatic effects by engaging the Integrin family and CD44 receptors, subsequently triggering intracellular cascades such as the PI3K/AKT and MAPK/ERK pathways [16]. For instance, previous studies have demonstrated its role in promoting epithelial-mesenchymal transition (EMT) in lung adenocarcinoma via macrophage-derived SPP1 upregulation of N-cadherin and vimentin; in hepatocellular carcinoma, SPP1 facilitates the formation of lung premetastatic niches through complex interactions among tumor cells, bronchial epithelium, and neutrophils [64, 65]. Similarly, in AM, elevated levels of SPP1, CCL2, and MMP9 were observed, promoting an M2 phenotype with anti-inflammatory properties and thereby contributing to an immunosuppressive TME [66–68]. Consistent with these established integrin-mediated mechanisms, our study identified the specific SPP1-ITGB5 axis as a driver of monocyte chemokine CCL2 expression, highlighting the potential role of SPP1^+^ macrophages in establishing an autocrine loop that sustains self-amplifying M2 macrophage reprogramming in LN^+^ AM.

Furthermore, recent single-cell and spatial transcriptomic investigations across multiple solid tumors have highlighted a profound role for SPP1^+^ macrophages in mediating resistance to immune checkpoint blockade (ICB) therapies [69, 70]. In ICB-resistant microenvironments, these specialized macrophages frequently localize to the tumor stroma boundaries, creating a dense physical and biochemical barrier that spatially restricts the infiltration of cytotoxic CD8^+^ T cells. In the context of AM, which is notoriously refractory to standard anti-PD-1/CTLA-4 regimens [71–73], the self-amplifying CCL2-driven self-amplifying SPP1^+^ macrophages likely accelerates the formation of this T-cell exclusion zone. By sustaining a continuously suppressive myeloid network, macrophage-derived SPP1 not only facilitates metastatic dissemination but also deeply compromises local antitumor immunity, providing a mechanistic rationale for the clinical observation that high SPP1 expression predicts poor immunotherapeutic responses.

However, the specific interactions between these macrophages and malignant melanoma cells have not been fully elucidated in prior studies. Through our deeper analysis of the SPP1 axis, we uncovered distinct interactions between SPP1^+^ macrophage and melanoma subclusters. Our study revealed macrophage-derived SPP1 sustained a pro-metastatic S100A8^+^ melanoma subcluster via the SPP1-CD44 pathway. CD44 is a well-documented transmembrane receptor heavily implicated in tumor stemness, cell-matrix adhesion, and resistance to apoptosis [74, 75]. By engaging CD44, macrophage-derived SPP1 likely activates profound cytoskeletal remodeling and survival signaling within the tumor cells. This explains why this specific subcluster, co-expressing CD44, dominated in advanced LN^+^ AM lesions (56.3%). While prior scRNA-seq studies have implicated interactions such as those between MYC^+^ tumor cells and FGFBP2^+^ NKT cells [9], our findings further complement these by pinpointing the SPP1-CD44 axis as a key driver of this S100A8^+^ subpopulation’s plasticity and invasive capacity. These results align perfectly with the established role of S100A8/A9 as a prognostic biomarker in melanoma [76, 77] and organotropic metastasis [78].

## Conclusions

In conclusion, our study identifies the SPP1-CD44 axis as a key driver of acral melanoma (AM) progression, promoting tumor cell plasticity via the expansion of the S100A8^+^ subpopulation, which is auto-amplified by CCL2-driven recruitment of monocytes in the LN^+^ TME. Clinically, high SPP1 expression in macrophages correlated with poorer survival, highlighting its potential as a robust prognostic indicator.

While our mechanistic findings are extensively supported by in vitro and in vivo experiments, several limitations should be acknowledged. First, the clinical cohort size remains relatively limited, which is largely inherent to the rarity of AM. Second, although our data support a coherent signaling framework linking SPP1–CD44 activation to downstream pro-invasive programs, direct experimental validation of each individual signaling branch warrants further investigation. Third, future spatially resolved analyses will be required to precisely map the anatomical co-localization and dynamic interactions of these cellular populations within human tissues. Nevertheless, disrupting the SPP1 axis might effectively suppress the tumor’s interactions with its immunosuppressive microenvironment, offering a promising avenue for future translational research.

## Availability of data and materials

The raw sequence data reported in this paper have been deposited in the Genome Sequence Archive in National Genomics Data Center with accession number HRA010713. The data supporting the findings of this study are included in the supplements of this paper. The source code used in this study is publicly available on GitHub at: https://github.com/zyl-0909/AM-scRNA-Seq.

## Supplementary information

Supplementary Table 1, Table 2, Table 3

Supplementary Figure

Supplement figure legends
