# Supplementary material for: SPP1+ macrophage-driven interactions shape the tumor microenvironment in lymph node metastatic acral melanoma
Source: Cell Death Dis. 2026 Apr 22;17(1):531. doi: 10.1038/s41419-026-08755-5 (PMC13230793; doi:10.1038/s41419-026-08755-5)
Supplement: Supplementary file 1 — Supplementary Table 1, Table 2, Table 3 [file 41419_2026_8755_MOESM1_ESM.docx]

| **Supplementary Table 1. Clinicopathological features of acral melanoma patients in scRNA-seq dataset.** | | | | | | | | | | | |
| --- | --- | --- | --- | --- | --- | --- | --- | --- | --- | --- | --- |
| **Sample** | **Sample type** | **Age**  **（years）** | **Gender** | **Depth of invasion (mm)** | **Ulceration** | **Tumor Stage** | **Lymph node** **metastasis** | **Stage^a^** | **Site of sample collection** | **CLARK level** | **Ki-67** |
| 540232T | tumor | 48 | female | 0 | Absence | Tis | LN^-^ | Tis | Left toe | NA | 20% |
| 541058T | tumor | 67 | male | 1.5 | Absence | T2 | LN^-^ | IB | Left toe | 3 | 20% |
| 556404T | tumor | 64 | female | 2 | Presence | T2 | LN^-^ | IIA | Right plantar | 4 | 10% |
| 281704T | tumor | 59 | female | 6 | Absence | T4 | LN^-^ | IIB | Right plantar | 5 | 60% |
| 539660T | tumor | 60 | male | 7.2 | Presence | T4 | LN^-^ | IIC | Left toe | 5 | 40% |
| 540585T | tumor | 62 | female | 6 | Presence | T4 | LN^-^ | IIC | Right plantar | 4 | 30% |
| 559901T | tumor | 99 | male | 4 | Presence | T3 | LN^+^ | IIIC | Right plantar | 4 | 35% |
| 599923T | tumor | 70 | male | 6 | Absence | T4 | LN^+^ | IIIC | Left plantar | 5 | 30+% |
| 563943T | tumor | 97 | male | 12 | Presence | T4 | LN^+^ | IIID | Right plantar | 4 | 90% |
| 591531N | normal | 72 | female | NA | NA | NA | LN^-^ | NA | Right heel adjacent normal tissue | NA | NA |
| 571157N | normal | 96 | female | NA | NA | NA | LN^+^ | NA | Left toe adjacent normal tissue | NA | NA |
| 599923N | normal | 70 | male | NA | NA | NA | LN^+^ | NA | Left plantar adjacent normal tissue | NA | NA |
| 563943N | normal | 97 | male | NA | NA | NA | LN^+^ | NA | Right plantar adjacent normal tissue | NA | NA |

**Abbreviations:** scRNA-seq, single cell RNA sequencing; NA, not available; LN^-^, lymph node non-metastasis; LN^+^, lymph node metastasis; ^a^ Eighth edition of the American Joint Committee on Cancer Staging Manual.

**Supplementary Table 2. Clinicopathologic features of acral melanoma patients undergoing immunofluorescence staining.**

| **No.** | **Site of sample collection** | **Gender** | **Age**  **（year）** | **Tumor maximum diameter (cm)** | **Ulceration** | **Clark level** | **Ki-67** | **Pathologic mitotic figures/mm²** | **Depth of invasion (mm)** | **Lymph node metastasis** |
| --- | --- | --- | --- | --- | --- | --- | --- | --- | --- | --- |
| A01 | Right plantar | Male | 70 | 3 | Presence | IV | 30%+ | 6 | 2 | LN^-^ |
| A03 | Plantar | Female | 63 | 3.5 | Presence | IV | 50%+ | 10 | 3 | LN^-^ |
| A07 | Right plantar | Male | 67 | 3.7 | Presence | V | 70%+ | 10 | 1.2 | LN^+^ |
| A09 | Right plantar | Male | 67 | 4.5 | Presence | IV | 20%+ | 4 | 4.3 | LN^-^ |
| A11 | Right plantar | Female | 40 | 2.5 | Presence | IV | 30%+ | 8 | 3.78 | LN^+^ |
| B03 | Left plantar | Female | 83 | 8 | Presence | IV | 20%+ | 5 | 4.8 | LN^+^ |
| B05 | Right plantar | Female | 69 | 5.7 | Presence | IV | 30%+ | 6 | 4.5 | LN^+^ |
| B07 | Right plantar | Male | 67 | 2 | Absence | IV | 40%+ | 10 | 5 | LN^+^ |
| B09 | Left plantar | Male | 60 | 4.5 | Presence | V | 60%+ | 30 | 13 | LN^+^ |
| C01 | Left plantar | Male | 62 | 4.5 | Presence | IV | 40%+ | 10 | 5.8 | LN^+^ |
| C03 | Right plantar | Female | 59 | 2.5 | Presence | IV | 5%+ | 2 | 1.5 | LN^+^ |
| C05 | Right plantar | Male | 66 | 1.6 | Presence | IV | 70%+ | 20 | 6.5 | LN^+^ |
| C09 | Right heel | Female | 75 | 2 | Absence | V | 5%+ | 3 | 3 | LN^-^ |
| C11 | Right index finger | Male | 61 | 2 | Presence | V | 40%+ | 2 | 1 | LN^-^ |
| C13 | Right plantar | Male | 81 | 1.6 | Absence | II | 40%+ | 2 | 2 | LN^-^ |
| D01 | Right plantar | Male | 48 | 4 | Absence | II | 10%+ | 1 | 1 | LN^-^ |
| D07 | Left plantar | Male | 58 | 2.5 | Presence | II | 50%+ | 3 | 2 | LN^-^ |
| D13 | Right plantar | Female | 32 | 2.5 | Absence | III | 40%+ | 4 | 0.22 | LN^-^ |
| E03 | Left plantar | Male | 63 | 2 | Presence | IV | 60%+ | 4 | 0.7 | LN^+^ |
| E09 | Left plantar | Female | 67 | 6 | Absence | II | 30%+ | 3 | 2 | LN^-^ |
| F05 | Left plantar | Female | 77 | 5 | absence | IV | 40%+ | 4 | 1.1 | LN^-^ |
| F07 | Right plantar | Female | 69 | 2.5 | Presence | V | 20%+ | 5 | 4.2 | LN^+^ |
| F09 | Right plantar | Female | 71 | 5 | Presence | IV | 40%+ | 10 | 4 | LN^-^ |
| G05 | Right thumb | Male | 62 | 3 | Presence | IV | 30%+ | 4 | 2.3 | LN^-^ |
| G11 | Right plantar | Male | 51 | 2.5 | Presence | IV | 60%+ | 8 | 1.9 | LN^+^ |

**Abbreviations:** LN^-^, lymph node non-metastasis; LN^+^, lymph node metastasis.

**Supplementary Table 3. Key Information and Sequence of the Mouse Spp1 shRNA Vector**

| **Item** | **Content** |
| --- | --- |
| Quote | VPSLV2SH251125CSD1-S-2 |
| Target gene | Mouse Spp1 |
| Method / vector construct | LP924-2: LV-U6>mSpp1[shRNA#2]-PGK>EGFP/T2A/Puro |
| shRNA identifier | shRNA #2 |
| shRNA sequence (5'-3') | TCCCGGTGAAAGTGACTGATTCTCGAGAATCAGTCACTTTCACCGGGA |
| Restriction enzyme validation | SacI: 4.5/3.7/0.2 kb; ApaLI: 5.1/2.0/1.2 kb; BamHI: 8.4 kb |

**Abbreviations:** shRNA, short hairpin RNA; EGFP, enhanced green fluorescent protein; T2A, Thosea asigna virus 2A peptide; PGK, phosphoglycerate kinase promoter; LV, lentiviral vector.

**Note:** The lentiviral knockdown vector targeting mouse Spp1 was constructed as LV-U6>mSpp1[shRNA#2]-PGK>EGFP/T2A/Puro. The shRNA sequence shown is the full hairpin oligonucleotide in the 5′–3′ direction. Fragment sizes listed for SacI, ApaLI, and BamHI represent the expected digestion products for vector validation.
