# Supplementary figures and images for: SPP1+ macrophage-driven interactions shape the tumor microenvironment in lymph node metastatic acral melanoma
Source: Cell Death Dis. 2026 Apr 22;17(1):531. doi: 10.1038/s41419-026-08755-5 (PMC13230793; doi:10.1038/s41419-026-08755-5)

Supplement Figure  
Fig.S1

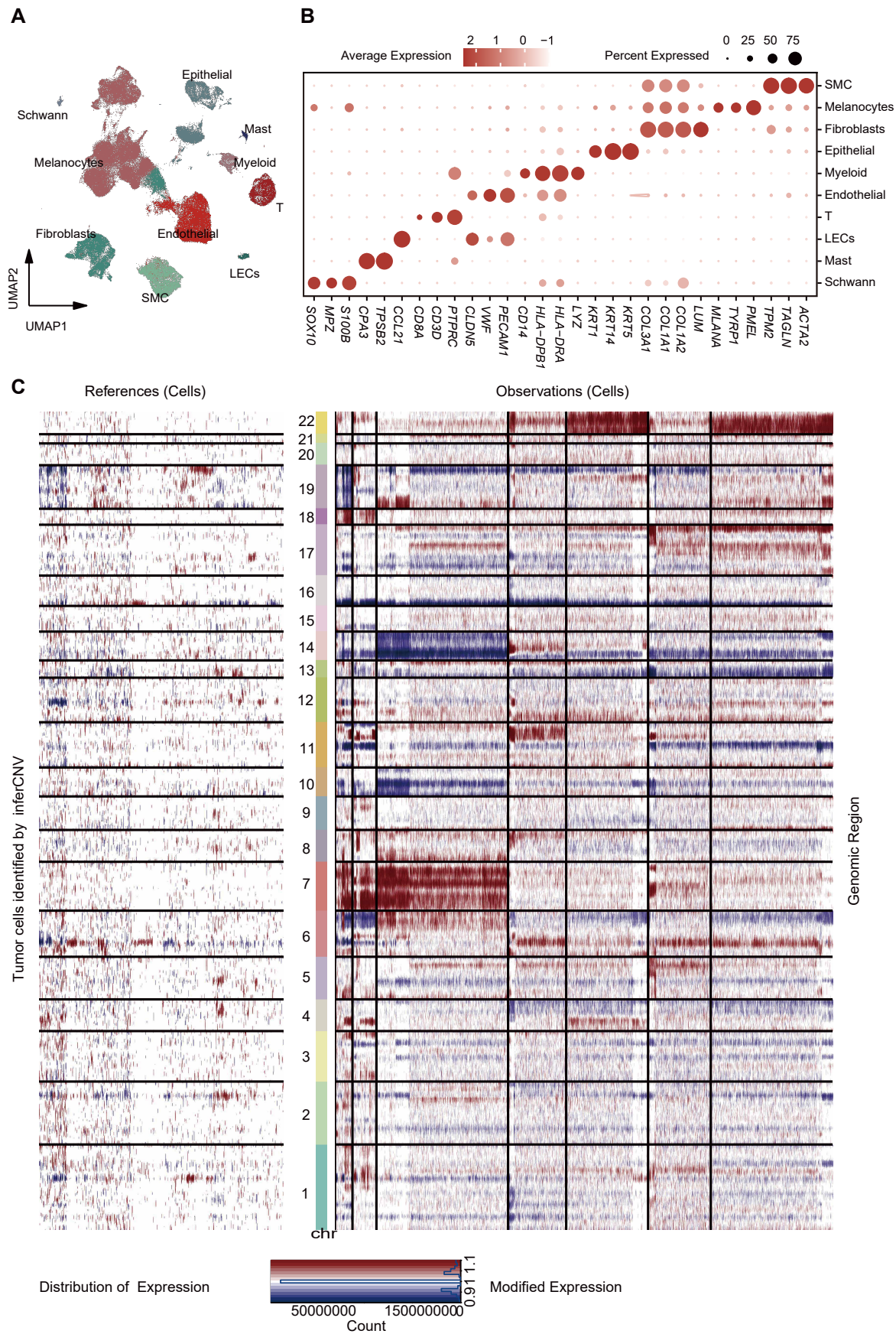

Fig.S2

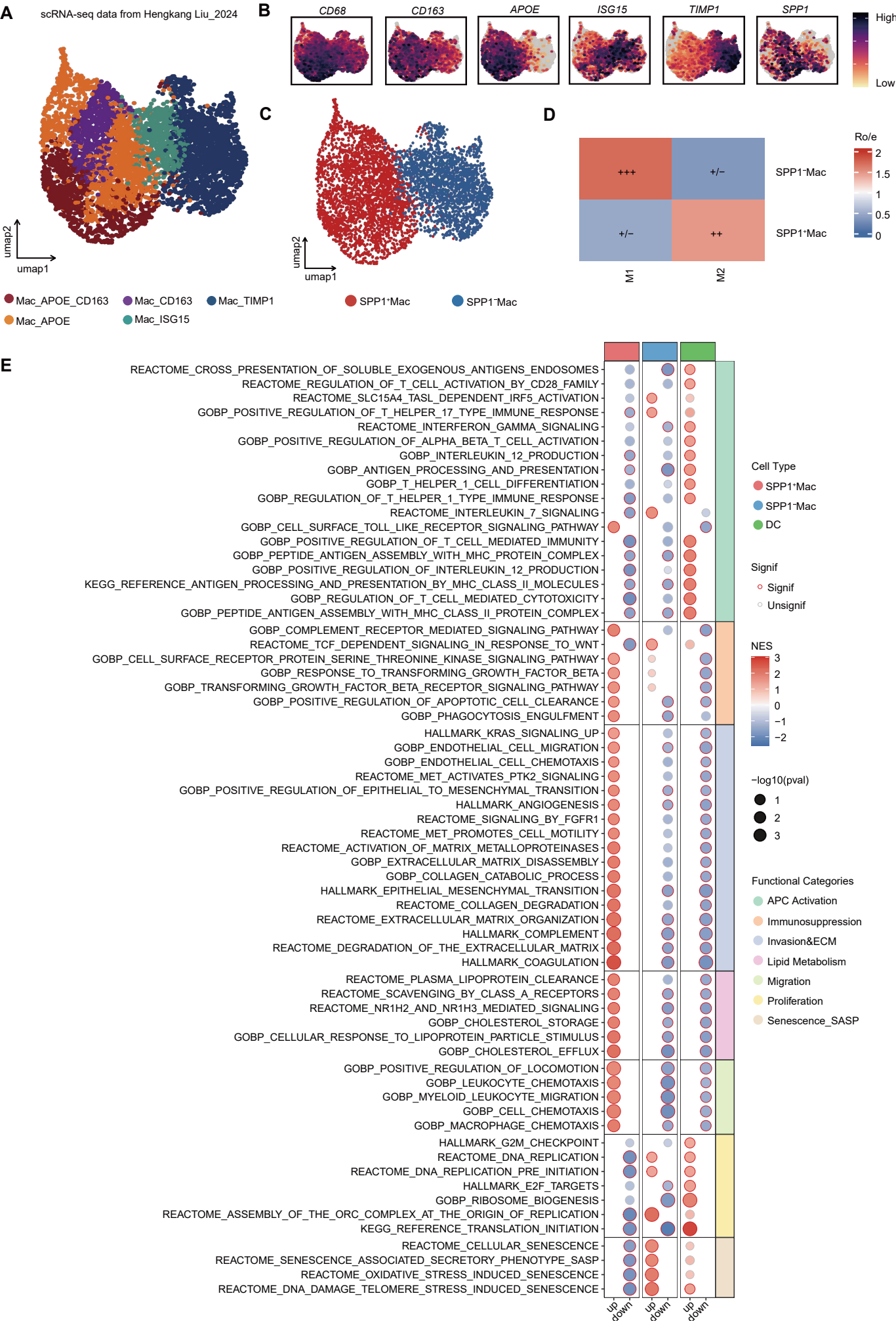

Fig.S3

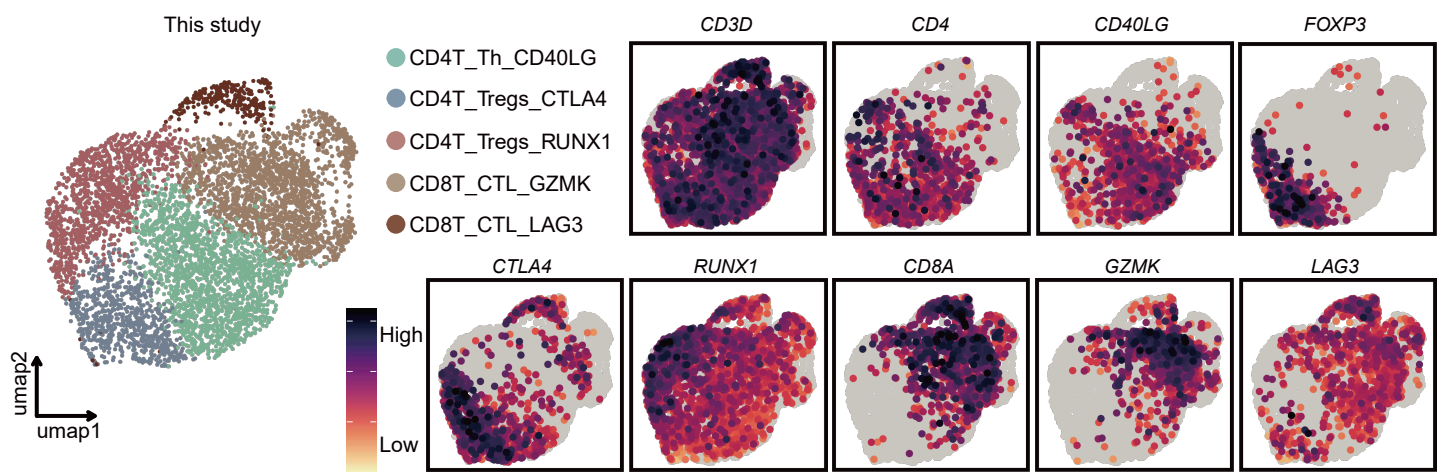

Fig.S4

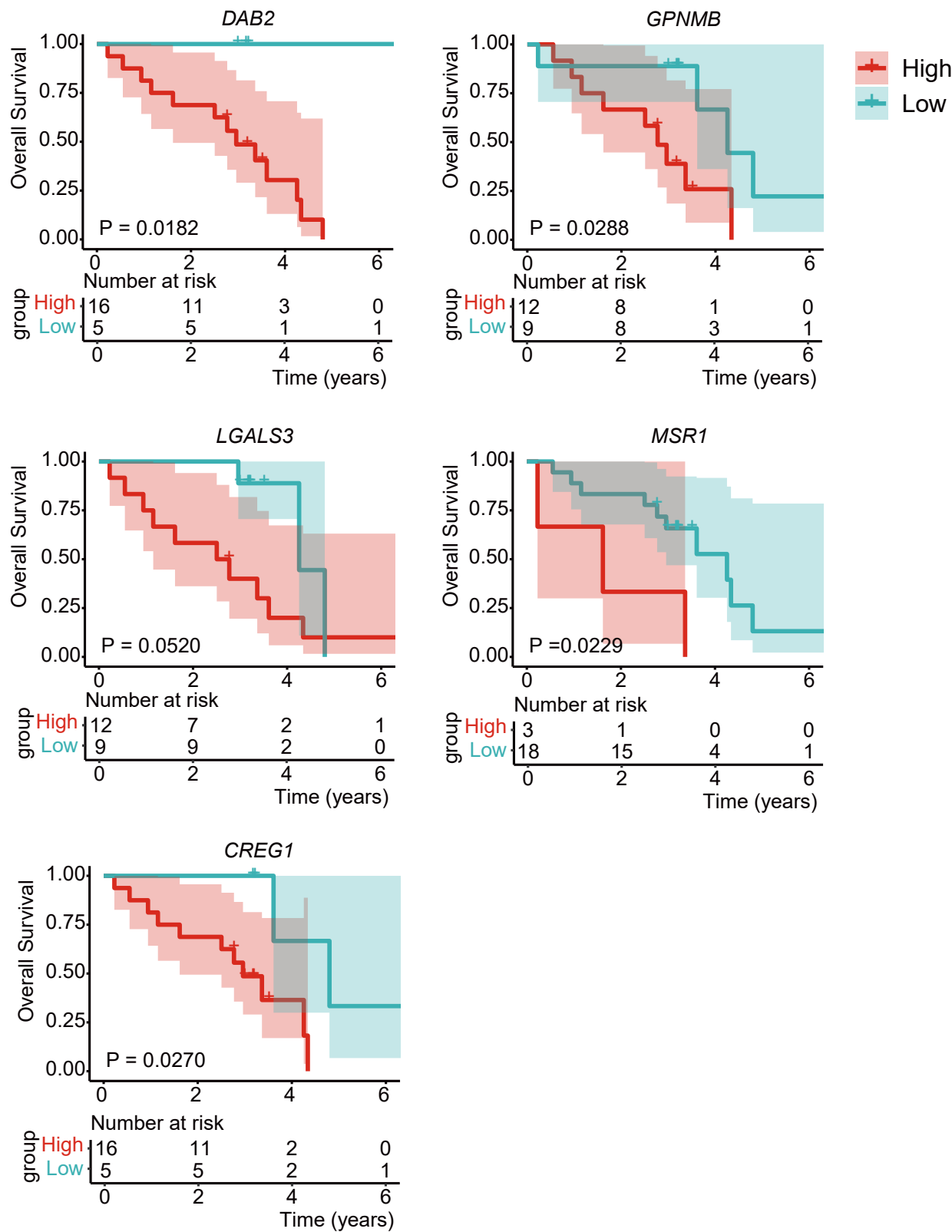

Fig.S5

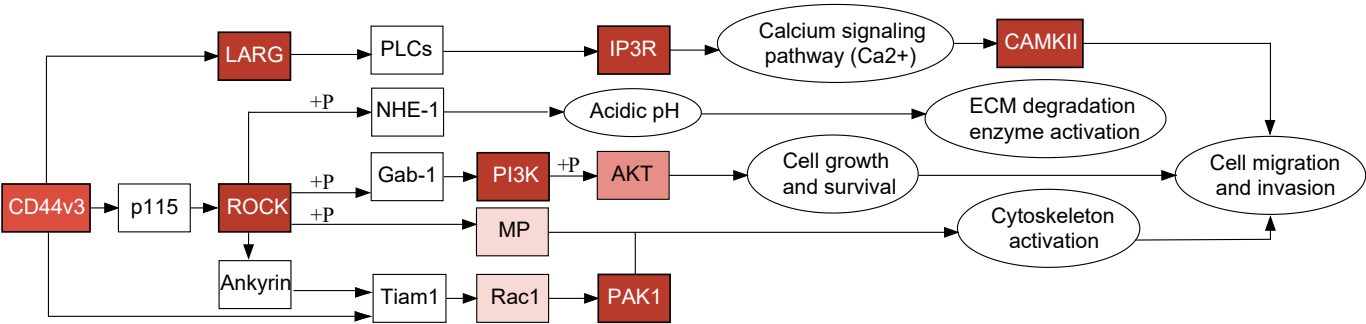

Supplement: Supplementary file 2 — Supplementary Figure [file 41419_2026_8755_MOESM2_ESM.pdf]
