# Supplementary material for: SPP1+ macrophage-driven interactions shape the tumor microenvironment in lymph node metastatic acral melanoma
Source: Cell Death Dis. 2026 Apr 22;17(1):531. doi: 10.1038/s41419-026-08755-5 (PMC13230793; doi:10.1038/s41419-026-08755-5)
Supplement: Supplementary file 3 — Supplement figure legends [file 41419_2026_8755_MOESM3_ESM.docx]

**Supplement figure legends**

**Figure S1. Annotation, and global intercellular communication analysis of the single-cell RNA-seq data.**

**(A)** UMAP plot demonstrating the cell distribution from primary AM tissues, color-coded by the annotated cell types.

**(B)** Dot plot identifying the expression of selected marker genes in the annotated cell types. Dot size: percentage of cells expressing the gene; dot color: scaled average expression.

**(C)** Copy number profile of tumor cells and reference cells (mast cells) determined by the inferCNV. Red, amplification; Blue, deletion.

**Figure S2. Heterogeneity of macrophage subpopulations and SPP1-associated polarization states.**

**(A)** UMAP visualization of macrophage subclusters from the external dataset. Cells are colored by subtype annotation, with **(B)** corresponding canonical markers listed.

**(C)** UMAP plot of the macrophage population, categorized into SPP1^+^ and SPP1^-^ subgroups based on SPP1 gene expression.

**(D)** The heatmap showing the macrophage polarization preference of macrophage subtypes, as determined by the ratio of observed to expected cell counts (Ro/e). Symbols in the heatmap correspond to the following Ro/e value ranges: +++ (Ro/e > 1.5), ++ (1 < Ro/e ≤ 1.5), + (0.5 ≤ Ro/e ≤ 1), +/− (0 < Ro/e < 0.5), and − (Ro/e = 0).

**(E)** Dot plot summarizing GSEA enrichment across SPP1^+^ macrophages, SPP1^-^ macrophages, and DCs. Pathways were grouped into functional categories, including Invasion & ECM remodeling, Immunosuppression, Lipid metabolism, APC activation, Proliferation, Senescence/SASP, and Migration. Each dot represents a pathway, with color indicating the normalized enrichment score (NES), size representing the –log10(p-value), and border color denoting statistical significance. SPP1^+^ macrophages show strong and selective enrichment of hallmark M2-like programs—particularly ECM remodeling, complement/coagulation, TGF-β–related immunosuppression, lipid metabolism, and chemotaxis—highlighting their pro-remodeling and immunoregulatory phenotype in LN^+^ tumors.

**Figure S3. Heterogeneity of T cell populations.**

UMAP visualization of T cell subclusters from this study. Cells are colored by subtype annotation, with corresponding canonical markers listed on the right.

**Figure S4. Prognostic significance of SPP1^+^ macrophage upregulated genes in AM.**

Kaplan-Meier survival curves depicting the prognostic significance of upregulated genes within SPP1^+^ macrophage cells, assessed in AM patients from the GSE162682 dataset. The analysis demonstrates that elevated expression of these genes is strongly associated with decreased overall survival, indicating their potential as markers of poor prognosis in AM.

**Figure S5. KEGG-based reconstruction of CD44 downstream signaling annotated with differentially expressed genes in S100A8^+^ melanoma cells (LN^+^ vs. LN^−^).**

Schematic overview of the CD44 signaling pathway annotated with differentially expressed genes identified in S100A8^+^ melanoma cells when comparing LN^+^ versus LN^-^ tumors. Red-shaded nodes represent genes upregulated in LN^+^ samples, indicating pathway activation. In LN^+^ tumors, S100A8^+^ melanoma cells display enhanced signaling through multiple CD44-mediated modules, including ROCK, LARG, PI3K–AKT, PAK1, IP3R, and CaMKII. These modules are associated with biological processes such as ECM remodeling, cytoskeletal regulation, cell migration, and invasion.
